# Silencing TMED2 suppresses cell growth and tumor progression in diffuse large B-cell lymphoma via inducing G0/G1 cell cycle arrest

**DOI:** 10.3389/fonc.2026.1810056

**Published:** 2026-05-01

**Authors:** Wei Qian, Mingzhen Yang

**Affiliations:** 1 Department of Hematology, The First Affiliated Hospital of Anhui Medical University, Hefei, Anhui, China; 2 Department of Hematology, Anhui Public Health Clinical Center, Hefei, Anhui, China

**Keywords:** diffuse large B-cell lymphoma, G0/G1 phase, therapeutic target, TMED2, xenograft model

## Abstract

**Background:**

Transmembrane Emp24 Domain Containing 2 (TMED2) is involved in various cancers, but its role in diffuse large B-cell lymphoma (DLBCL) remains unclear. This study investigated TMED2’s expression, biological functions, and underlying mechanisms in DLBCL.

**Methods:**

TMED2 expression was analyzed in DLBCL patient samples and cell lines by qRT-PCR and Western blot (WB). Lentiviral shRNA-mediated knockdown of TMED2 was performed in SUDHL-4 and OCI-LY10 DLBCL cells. Functional impacts on proliferation, cell cycle, and apoptosis were assessed using CCK-8, flow cytometry analysis, annexin V-APC staining assays, caspase-3/7 activity assays, and WB of key regulators (cyclins, CDKs, Bax, Bcl-2, caspases). The *in vivo* role of TMED2 was evaluated using a subcutaneous xenograft model in nude mice.

**Results:**

TMED2 expression was significantly upregulated in DLBCL tissues and cell lines. TMED2 knockdown markedly inhibited cell proliferation *in vitro*. This was associated with a pronounced G0/G1 phase cell cycle arrest, coupled with downregulation of key G1/S transition regulators (cyclin D1, cyclin E1, CDK2, CDK4, CDK6). Furthermore, TMED2 silencing promoted apoptosis, evidenced by increased Annexin V-positive cells, elevated caspase-3/7 activity, upregulated expression of cleaved caspase-3/caspase-7, and an increased Bax/Bcl-2 ratio. *In vivo*, TMED2 knockdown significantly suppressed tumor growth in xenograft models.

**Conclusion:**

TMED2 promotes DLBCL progression by driving cell cycle progression and inhibiting apoptosis. Silencing TMED2 induces G0/G1 arrest and enhances caspase-dependent apoptosis. These findings identify TMED2 as a potential prognostic biomarker and therapeutic target in DLBCL.

## 1 Introduction

Diffuse large B-cell lymphoma (DLBCL) represents the most prevalent subtype of non-Hodgkin lymphoma in adults, accounting for approximately 35% of all newly diagnosed cases (1, 2). Although immunochemotherapy, particularly the R-CHOP regimen (rituximab, cyclophosphamide, doxorubicin, vincristine, and prednisone), has markedly improved patient outcomes, approximately one-third of patients still experience primary refractory disease or relapse, contributing significantly to mortality rates (3). This clinical challenge is closely linked to the profound molecular and genetic heterogeneity of DLBCL, which underlies diverse treatment responses and clinical outcomes observed among patients. Advances in high-throughput genomic and transcriptomic profiling have enabled refined molecular classifications, including the cell-of-origin subtypes, germinal center B-cell-like (GCB) and activated B-cell-like (ABC), with the latter typically associated with inferior prognosis (4–6). Further stratification has identified genetic subtypes such as MCD, BN2, N1, and EZB, characterized by distinct mutational patterns involving B-cell receptor (BCR) signaling, NF-κB activation, and epigenetic regulators like EZH2 and CREBBP (7).

A hallmark of DLBCL pathogenesis is the persistent activation of proliferative and anti-apoptotic signaling pathways. Among these, the phosphoinositide 3-kinase/protein kinase B/mammalian target of rapamycin (PI3K/Akt/mTOR) axis plays a central role in regulating cell growth, metabolism, and survival. In DLBCL, dysregulation of this pathway, commonly caused by PTEN loss or upstream mutations, often heralds an adverse prognosis, manifesting as advanced disease, elevated International Prognostic Index (IPI) scores, and diminished survival (8). Preclinical studies confirm that inhibiting this pathway induces tumor cell death, underscoring its therapeutic relevance (9). Concurrently, evasion of apoptosis is facilitated by alterations in Bcl-2 family proteins, including recurrent translocations and amplifications, as well as disruptions in the p53 tumor suppressor network (10, 11). The interplay among these dysregulated pathways fosters a permissive environment for lymphomagenesis, highlighting the need to identify additional molecular drivers, especially in high-risk and treatment-resistant cases.

In the search for novel therapeutic targets, attention has turned to proteins involved in fundamental cellular processes that may be co-opted in malignancy. Transmembrane Emp24 Domain Containing 2 (TMED2), a member of the p24 protein family, has recently emerged as a protein of interest in cancer biology. Localized primarily to the Golgi apparatus and endoplasmic reticulum (ER), TMED2 mediates vesicular protein trafficking, contributing to cargo selection, glycosylation, and secretion, processes essential for cellular homeostasis; Beyond this canonical role, TMED2 is frequently dysregulated in malignancies such as glioma, ovarian cancer, and breast cancer, where its overexpression correlates with aggressive phenotypes and poor prognosis (12). Mechanistically, TMED2 appears to function as a signaling modulator: in epithelial ovarian cancer, it promotes cell proliferation, migration, and invasion via the IGF2/IGF1R/PI3K/Akt axis (13), while in oral cancer, it was further discovered that the stemness and tumorigenicity of OC cells are potentiated by TMED2 via its direct control over GALNT7 expression (14). These findings position TMED2 as a multifunctional protein capable of engaging established oncogenic pathways.

Despite these insights in solid tumors, the role of TMED2 in hematologic malignancies, particularly DLBCL, remains largely unexplored. Our preliminary data provide the first evidence of its involvement in DLBCL pathogenesis. We observed high TMED2 expression in DLBCL cell lines such as SUDHL-4 and OCI-LY10. Functional studies demonstrated that TMED2 knockdown significantly suppresses proliferation, induces cell cycle arrest, particularly in G0/G1 phase, and promotes apoptosis *in vitro*. These findings were further supported by *in vivo* xenograft models, where TMED2 silencing significantly inhibited tumor growth. These results establish TMED2 as a promoter of DLBCL proliferation and survival. Unraveling the role of TMED2 will not only advance our understanding of DLBCL biology but may also identify it as a novel prognostic marker or therapeutic target, offering new avenues for intervention in this heterogeneous and challenging disease.

## 2 Materials and methods

### 2.1 Cell culture and treatment

Human DLBCL cell lines, including SUDHL4, OCI-LY10, OCI-LY19, and U2932, along with the immortalized normal B-cell line HMy2.CIR, were obtained from the Cell Bank of the Chinese Academy of Sciences located in Shanghai, China. DLBCL cell lines were cultured in RPMI-1640 medium (Hyclone, USA), and HMy2.CIR cells were grown in IMDM (Gibco) supplemented with 10% fetal bovine serum (FBS, Gibco, USA), 1% streptomycin, and penicillin (Hyclone, United States) in an incubator (ThermoFisher, United States) with 5% CO_2_ at 37 °C.

### 2.2 Clinical patient samples

A total of 17 diffuse large B-cell lymphoma (DLBCL) tissue samples and 12 reactive hyperplasia lymph node tissue samples were collected from patients diagnosed at the First Affiliated Hospital of Anhui Medical University between 5 March, 2022 and 12 March, 2024. None of the patients had received chemotherapy or radiotherapy prior to sample collection. Upon surgical resection or biopsy, the fresh tissue samples were immediately snap-frozen in liquid nitrogen and stored at -80 °C until subsequent total RNA and protein extraction. This study was conducted in accordance with the Declaration of Helsinki and was formally reviewed and approved by the Ethics Committee of Anhui Medical University (Approval Number:2022259). Written informed consent was obtained from all participating patients prior to the collection of clinical specimens.

### 2.3 qRT-PCR analysis

Total RNA was extracted from SUDHL-4 and OCI-LY10 cell lines using a commercially available RNA purification kit (New Cell & Molecular Biotech Co., Ltd., China). RNA purity and concentration were assessed on a NanoDrop 2000 spectrophotometer (Thermo Fisher Scientific, USA), and all samples showed an A260/A280 ratio within the acceptable range of 1.8 to 2.0. The purified RNA (2 µg) was reverse-transcribed into cDNA using the RevertAid cDNA Synthesis Kit (Vazyme Co., Ltd., China) according to the manufacturer’s instructions. Quantitative polymerase chain reaction was performed in a 25 µL reaction system containing UltraSYBR Green master mix (Vazyme Co., Ltd., China) on a LightCycler^®^ 96 instrument (Roche, Switzerland). Furthermore, for the 25 µL qPCR reaction system, we have clarified that it contained 2 µL of cDNA template and a final primer concentration of 5 µM. GAPDH was used as the endogenous reference gene for normalization, and relative gene expression was calculated via the comparative Ct (2^−ΔΔCt)^ method. The primer sequences used in the amplification are listed below:GAPDH forward: TGACTTCAACAGCGACACCCA, GAPDH reverse: CACCCTGTTGCTGTAGCCAAA; TMED2 forward: GGTGACGCTTGCTGAACTGC, TMED2 reverse: AGATGAGGCCCATCTTGGTG.

### 2.4 Western blot analysis

As previously described (15, 16), total protein was extracted from the cells using RIPA lysis buffer (Thermo Fisher Scientific, Inc.) supplemented with a protease and phosphatase inhibitor cocktail (Roche Diagnostics). The protein concentration of each sample was quantified using a BCA Protein Assay kit (Beyotime Institute of Biotechnology). The protein (20 µg per lane) were resolved by 10% sodium dodecyl sulfate-polyacrylamide gel electrophoresis (SDS-PAGE) and subsequently electrotransferred onto polyvinylidene difluoride (PVDF) membranes (EMD Millipore). Then, the membranes were blocked with 5% non-fat milk in Tris-buffered saline with Tween-20 (TBST) for 1 h at room temperature. Following blocking, the membranes were incubated overnight at 4 °C with specific primary antibodies. The primary antibodies used in this study were as follows:

Antibodies purchased from Proteintech included: Cyclin D1 (cat. no. 60186-1-Ig, dilution: 1:5000), Cyclin E1 (cat. no. 11554-1-AP, dilution: 1:2000), CDK2 (cat. no. 10122-1-AP, dilution: 1:5000), Cyclin A1 (cat. no. 13295-1-AP, dilution: 1:1000), CDK4 (cat. no. 66950-1-Ig, dilution: 1:5000), Cyclin B1 (cat. no. 67686-1-Ig, dilution: 1:5000), CDK1 (cat. no. 84271-1-RR, dilution: 1:5000), CDK6 (cat. no. 14052-1-AP, dilution: 1:2000), and β-actin (cat. no. 66009-1-Ig, dilution: 1:20000). Antibodies obtained from Cell Signaling Technology (CST) included: Bax (cat. no. 2772, dilution: 1:1000), Bcl-2 (cat. no. 3498, dilution: 1:1000), Cleaved Caspase-3 (cat. no. 9664, dilution: 1:1000), Caspase-3 (cat. no. 9662, dilution: 1:1000), and Caspase-7 (cat. no. 9492, dilution: 1:1000), and TMED2 (cat. no.ab129412, dilution: 1:300) was purchased from Abcam.

After washing with TBST, the membranes were incubated with horseradish peroxidase (HRP)-conjugated secondary antibodies (Anti-Mouse IgG (CST, #7076, dilution: 1:2000) or Anti-Rabbit IgG (CST, #7074, dilution: 1:2000) for 1 h at room temperature. Dilute the primary antibody and secondary antibody with 5% BSA or 5% non-fat milk in TBST, respectively. The protein bands were visualized using Western Bright™ ECL reagent (Advansta, Inc.) and imaged using the ChemiDoc MP system (Bio-Rad Laboratories, Inc.). Band intensity was quantified using Image J software (version 1.8.0; National Institutes of Health). All experiments were performed in triplicate.

### 2.5 Lentivirus production and infection

The lentiviral plasmid hU6-MCS-CMV-EGFP purchased from Shanghai Genechem Co., Ltd. (Shanghai, China) were used for shRNA-mediated knockdown in DLBCL cells. Select the mRNA of Homo sapiens transmembrane p24 trafficking protein 2 (TMED2), transcript variant 1 as the target gene for infection. The target sequence for RNA interference is ATGGATGGAACATACAAAT. Additionally, empty vectors of shCtrl was used as negative control. The DLBCL cell lines were transfected with the constructed lentiviral plasmids for 72 h, followed by fluorescence observation and photographic documentation under a microscope. Following a 5-day transfection period, the cells were subsequently utilized for further analysis. DLBCL cells were infected with lentiviral vectors at specific MOIs (20 for SUDHL-4; 50 for OCI-LY10). At 72 h post-infection, fluorescence microscopy confirmed an infection efficiency exceeding 80% for both cell lines. Given this high baseline efficiency, functional assays were performed directly on these enriched populations to minimize potential phenotypic drift from prolonged antibiotic selection. Knockdown efficiency was rigorously verified via qRT-PCR and Western blot prior to all downstream experiments.

### 2.6 Cell proliferation assay

Cell viability was assessed with a Cell Counting Kit-8 (CCK-8) assay following an established protocol (17, 18). Briefly, after being transduced with shRNA lentivirus, DLBCL cells were cultured for 5 days to allow for stable knockdown. Subsequently, the cells were seeded in a 96-well plate at a density of 1 × 10^6^ cells/mL, with 100 µL per well. Blank wells (medium only) and control wells (cells transduced with shCtrl) were included, and each experimental condition was performed in triplicate. Following the addition of 10 µL of CCK8 reagent (Kumamoto, Kyushu, Japan) to each well, the plate was incubated at 37 °C for 2 h. Absorbance was then measured at 450 nm using a microplate reader (ThermoFisher, USA). Cell proliferation was calculated according to the following formula:

Cell proliferation (%) = [OD (test) – OD (blank)]/[OD (control) – OD (blank)] × 100%.

The fold change in absorbance at 450 nm over time was compared between the shTMED2 group and the shCtrl group.

### 2.7 Cell cycle analysis

As previously described (19), cell cycle distribution was analyzed in OCI-LY10 and SUDHL-4 cells following a 5-day treatment with either shCtrl or shTMED2. Briefly, cells were harvested by trypsinization, washed with phosphate-buffered saline (PBS), and fixed in 70% ice-cold ethanol overnight at −4 °C. Following fixation, the cells were rinsed three times with PBS and then incubated in the dark at 37 °C for 30 min with a staining solution prepared at a ratio of 40×PI (2 mg/mL): 100×RNase (10 mg/mL): 1×D-Hanks = 25:10:1000. According to the cell quantity, resuspend the cells by adding an appropriate volume of cell staining solution (0.6–1 mL) to achieve a cell flow rate of 300–800 cells per second during flow cytometry analysis. Cell cycle analysis was performed using a BD FACScan flow cytometer (BD Biosciences) with an excitation wavelength of 488 nm, and red fluorescence emission was collected. Data were analyzed to determine the percentage of cells in the G0/G1, S, and G2/M phases.

### 2.8 Measurement of cell apoptosis by flow cytometry

To assess apoptosis, the cells were stained with Annexin V-APC using a commercial apoptosis detection kit (88-8007, eBioscience), following the manufacturer’s instructions. As previously described (20), the cells (5 × 10^4^/well) were seeded in 6-well plates. After 72 h of incubation, harvest cells ensuring at least 5×10^5^ cells per group with a minimum of three replicate wells. Centrifuge at 1300 rpm for 5 min, discard supernatant, and wash cell pellet with pre−chilled PBS at 4 °C. Wash once with 1× binding buffer, centrifuge at 1300 rpm for 3 min, and collect cells. Resuspend the pellet in 200 μL of 1× binding buffer. Add 10 μL Annexin V−APC, incubate at room temperature protected from light for 10−15 min. Then, add 400−800 μL of 1× binding buffer according to cell density, and proceed to flow cytometry analysis. Samples were analyzed using flow cytometry (C6 PLUS, Becton Dickinson, USA). The experiment was independently repeated in triplicate, with results presented as mean ± standard deviation.

### 2.9 Caspases-3 and -7 activity assay

As previously described (21), Caspase-3/7 activity was assessed using the Homogeneous Caspase-Glo^®^ 3/7 Assay Kit (Promega, Madison, WI, USA), following the manufacturer’s protocol. Briefly, the SUDHL-4 and OCI-LY10 cells (1 × 10^3^ per well) were seeded into white-walled 96-well plates and incubated at 37 °C with 5% CO_2_ for 3–5 days to allow for adherence. After collecting the cells and performing cell counting, add 100 μl of the cell suspension (containing 1×10^4^ cells per well) to each well of the 96-well plate. Then, add 100 μl of Caspase-Glo reagent to each well. A blank control group without cells should also be set up, with only 100 μl of culture medium added per well. The plates were shaken and mixed gently at 300–500 rpm for 30 minutes and then incubated at room temperature for 2 h. Following this, luminescence was captured using an Olympus fluorescence microscope (IX71, Japan). Background luminescence was determined using a blank containing culture medium with DMSO (the vehicle for the test compounds), while untreated cells were included as a negative control.

### 2.10 Tumor xenograft model

Male BALB/c nude mice (aged 8–10 weeks, weighing 20–25 g) were purchased from the GemPharmath Co., LTD (Nanjing, China). Mice were kept in specific pathogen-free conditions with a 12-h light/dark cycle and were given unrestricted access to a certified standard diet and tap water. The mice were randomly allocated to two groups (6 per group): the shCtrl group and the shTMED2 group. For the construction of subcutaneous xenograft models, SUDHL-4 cells stably transduced with either shCtrl or shTMED2 vectors were resuspended in serum-free medium and mixed with Matrigel basement membrane matrix (BD Biosciences) at a 1:1 ratio (final volume 200 µl). A total of 5×10^6^ cells in this suspension were administered via subcutaneous injection into the right flank of each mouse (22). Tumor size was monitored every seven days with a vernier caliper. Tumor volume was determined using the formula: volume (mm³) = (width² × length)/2.

After 37 days, euthanasia was carried out via a 5% or higher isoflurane overdose, with administration maintained until one minute after the cessation of breathing. Cervical dislocation was subsequently performed to confirm death. Tumor tissues were harvested for subsequent experiments. Tumor growth curves were plotted, and the collected tissues were weighed. Animal experiments were approved by the Experimental Animal Ethics Committee of Anhui Medical University (Approval Number: LLCS20200786).

### 2.11 Statistical analysis

The statistical analyses in this study were performed with SPSS 26.0 (SPSS Inc., Chicago, IL, USA). Continuous data from at least three independent replicates are expressed as mean ± standard deviation. Statistical significance between shCtrl and shTMED2 groups were analyzed using Student’s t-test. Other comparisons were performed using one-way ANOVA followed by the Dunnett’s multiple comparison test. Overall survival was evaluated by Kaplan-Meier analysis, and differences were assessed with the log-rank test. A P-value less than 0.05 was regarded as statistically significant.

## 3 Result

### 3.1 The expression of TMED2 was upregulated in DLBCL patients and cell lines

**Figure 1:**
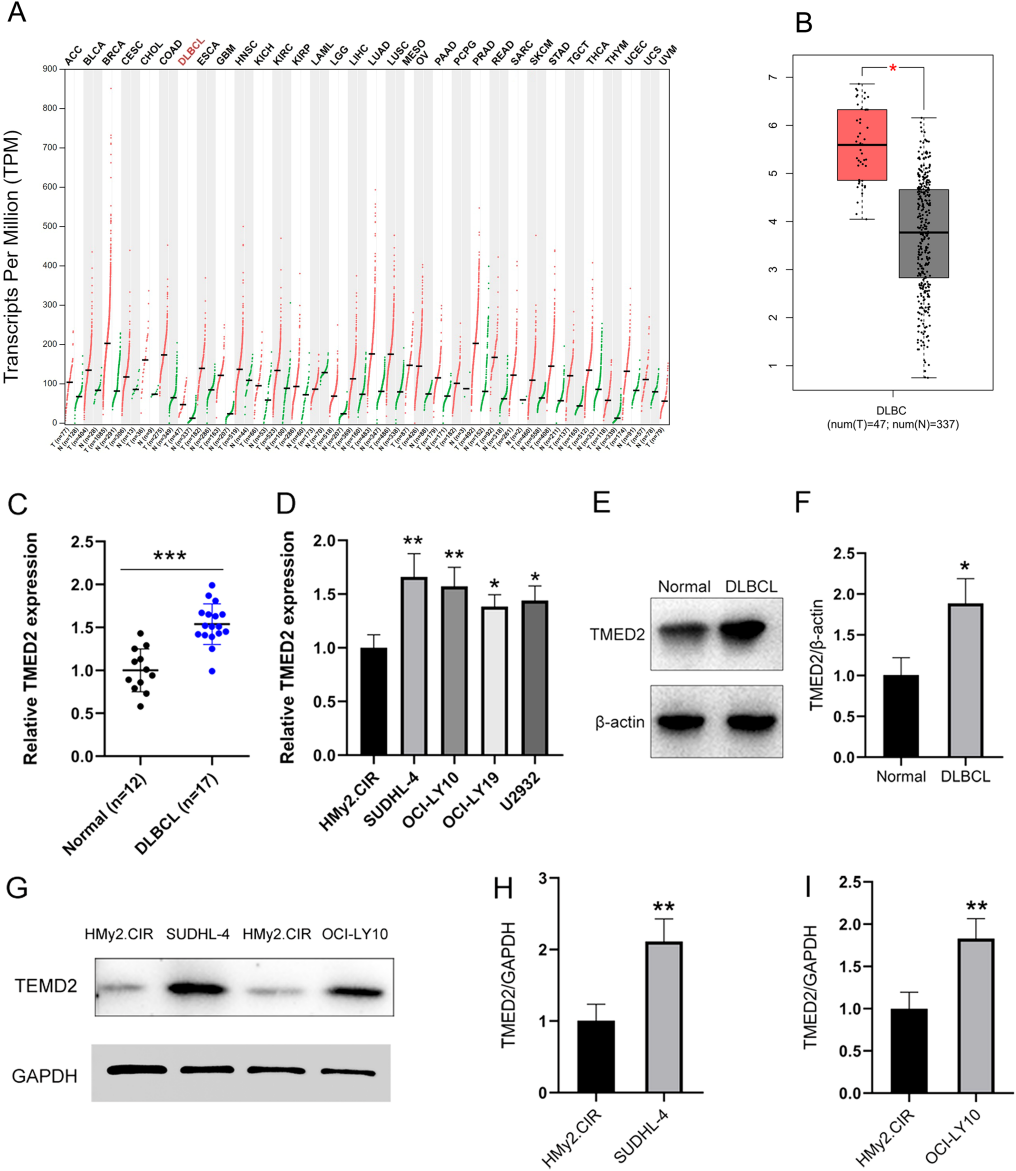
TMED2 expression is upregulated in DLBCL. **(A)**. Expression patterns of TMED2 in tumors and normal tissues from the GTEx and TCGA databases. ACC, Adenoid cystic carcinoma; BLCA, Bladder cancer; BRCA, Breast cancer; CESC, Cervical squamous cell carcinoma; CHOL, Cholangiocarcinoma; COAD, Colon adenocarcinoma; ESCA, Esophageal carcinoma; GBM, Glioblastoma; HNSC, Head and Neck Squamous Cell Carcinoma; KICH, Chromophobe Renal Cell Carcinoma; KIRC, Kidney Renal Clear Cell Carcinoma; KIRP, Kidney Renal Papillary Cell Carcinoma; LAML, Acute Myeloid Leukemia; LGG, Low-Grade Glioma; LIHC, Liver Hepatocellular Carcinoma; LUAD, Lung Adenocarcinoma; LUSC, Lung squamous cell carcinoma; MESO, Mesothelioma; OV, Ovarian cancer; PAAD, Pancreatic adenocarcinoma; PCPG, Paraganglioma and Pheochromocytoma; PRAD, Prostatic adenocarcinoma; READ, Rectal adenocarcinoma; SARC, Sarcoma; SKCM, Skin cutaneous melanoma; STAD, Stomach adenocarcinoma; TGCT, Tenosynovial giant cell tumor; THCA, Thyroid carcinoma; THYM, Thymic carcinoma; UCEC, Uterine corpus endometrial carcinoma; UCS, Uterine carcinosarcoma; UVM, Uveal melanoma. **(B)** Evaluation of TMED2 expression in DLBCL samples from the GTEx and TCGA databases. Red represents tumor (T) tissue, grey represents normal tissue (N). **(C)** mRNA expression levels of TMED2 in DLBCL patient tissues compared to normal controls. **(D)** mRNA expression of TMED2 in DLBCL cell lines (SUDHL-4, OCI-LY10, OCI-LY19, U2932) and the normal B-cell line HMy2.CIR. N = 3. **(E, F)** WB images and quantitative analysis of TMED2 protein expression in DLBCL patient tissues versus normal tissues. N = 3. **(G–I)** WB analysis and quantification of TMED2 protein levels in SUDHL-4 and OCI-LY10 cells. N = 3. *p < 0.05, **p < 0.01, ***p < 0.001.

First, analysis using the Gene Expression Profiling Interactive Analysis (GEPIA) website revealed that the transcriptional level of the TMED2 gene is elevated in most common tumors, including in DLBCL (**Figures 1A, B**). Furthermore, analysis of TMED2 expression in DLBCL patient tissues confirmed a significant upregulation at both the mRNA and protein levels (**Figures 1C, E, F**). This pattern was consistently observed in established DLBCL cell lines, which also exhibited elevated TMED2 mRNA (**Figure 1D**). Further investigation into specific cell lines, SUDHL-4 and OCI-LY10, confirmed that the increase in mRNA translated to a corresponding and significant rise in TMED2 protein expression (**Figures 1G–I**). Collectively, these comprehensive data from tissues, cell lines, and patient outcomes strongly suggest that TMED2 upregulation is a recurrent feature in DLBCL.

### 3.2 Knockdown of TMED2 suppressed cell proliferation in DLBCL cells

**Figure 2:**
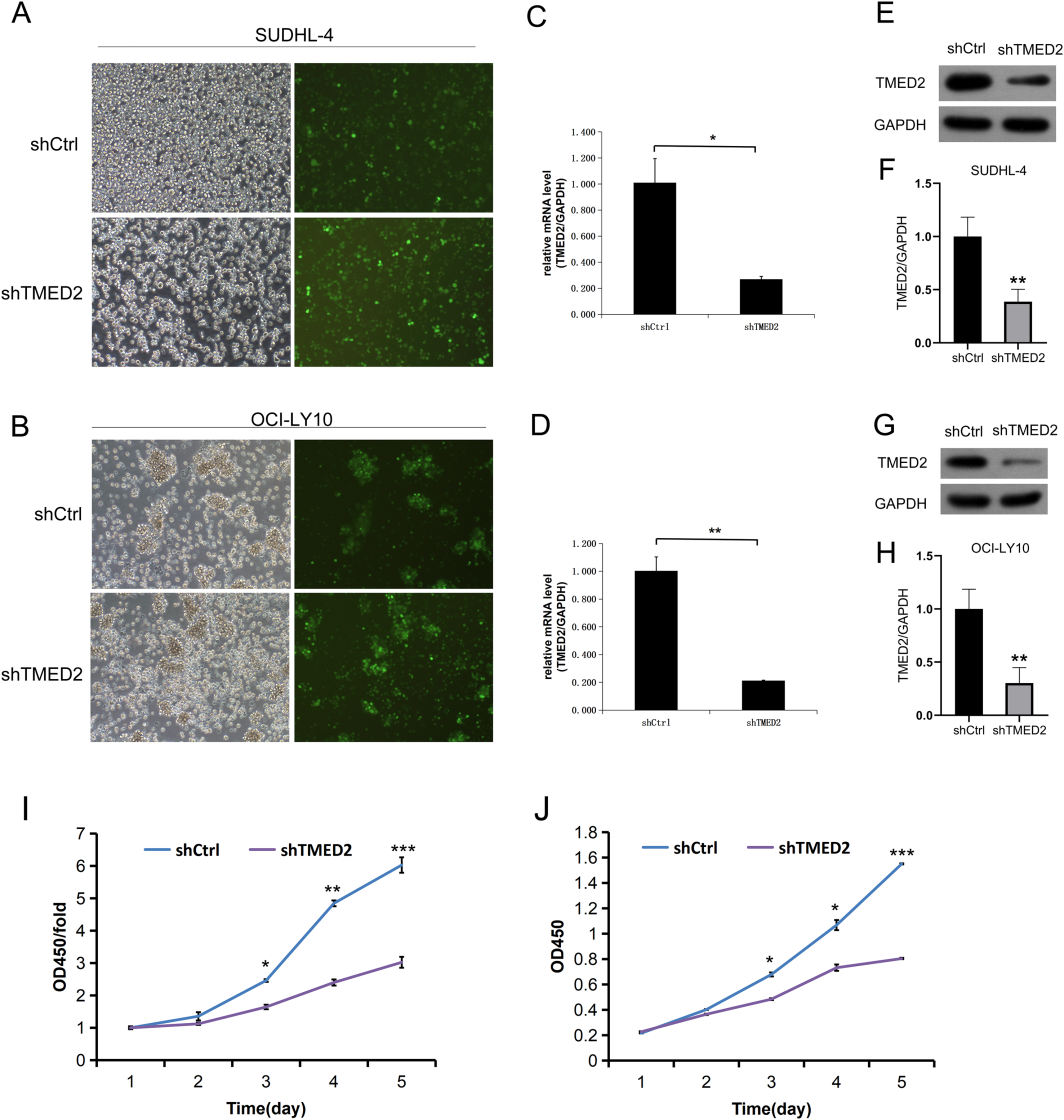
TMED2 knockdown suppresses cell proliferation in DLBCL cells. **(A, B)** Fluorescence microscopy images showing successful lentiviral transduction (green fluorescence) in SUDHL-4 **(A)** and OCI-LY10 **(B)** cells after 72 h of infection with shTMED2 or shCtrl. Magnification: 100x. **(C, D)** qRT-PCR analysis of TMED2 mRNA expression in SUDHL-4 **(C)** and OCI-LY10 **(D)** cells following TMED2 knockdown. **(E–H)** WB analysis of TMED2 protein expression in SUDHL-4 and OCI-LY10 cells after TMED2 knockdown. Representative blots **(E, G)** and densitometric quantification normalized to GAPDH **(F, H)** are shown. **(I, J)** Cell proliferation was measured using a CCK-8 assay in SUDHL-4 **(I)** and OCI-LY10 **(J)** cells over 5 days after TMED2 knockdown. All experiments were performed in triplicate. *p < 0.05, **p < 0.01, ***p < 0.001.

Based on our findings that TMED2 expression is elevated in both DLBCL patients and cell lines, we proceeded to investigate the functional impact of TMED2 knockdown in DLBCL cells. Following 72 h of lentiviral infection targeting TMED2 or a non-targeting control (shCtrl) in SUDHL-4 and OCI-LY10 cells, fluorescence microscopy revealed that infection efficiency exceeded 80% in both cell lines, with cells maintaining normal morphology (**Figures 2A, B**). Subsequent qRT-PCR analysis confirmed a significant reduction in TMED2 mRNA levels upon TMED2 knockdown in SUDHL-4 (p = 0.019, **Figure 2C**) and OCI-LY10 cells (p = 0.005, **Figure 2D**). Consistent with this, western blot analysis demonstrated a marked decrease in TMED2 protein expression in both cell lines following TMED2 knockdown (**Figures 2E–H**). Functional assessment using a CCK-8 proliferation assay revealed that TMED2 knockdown significantly inhibited the viability of SUDHL-4 and OCI-LY10 cells, with the most pronounced suppression observed on day five post-knockdown (**Figure 2I, J**). Taken together, these results indicate that TMED2 is effectively silenced at both transcriptional and translational levels in DLBCL cells, and its knockdown leads to a substantial impairment of cell proliferation, supporting a critical role for TMED2 in promoting DLBCL cell growth.

### 3.3 Knockdown of TMED2 inhibited cell growth by inducing cell cycle arrest at G0/G1 in DLBCL cells

**Figure 3:**
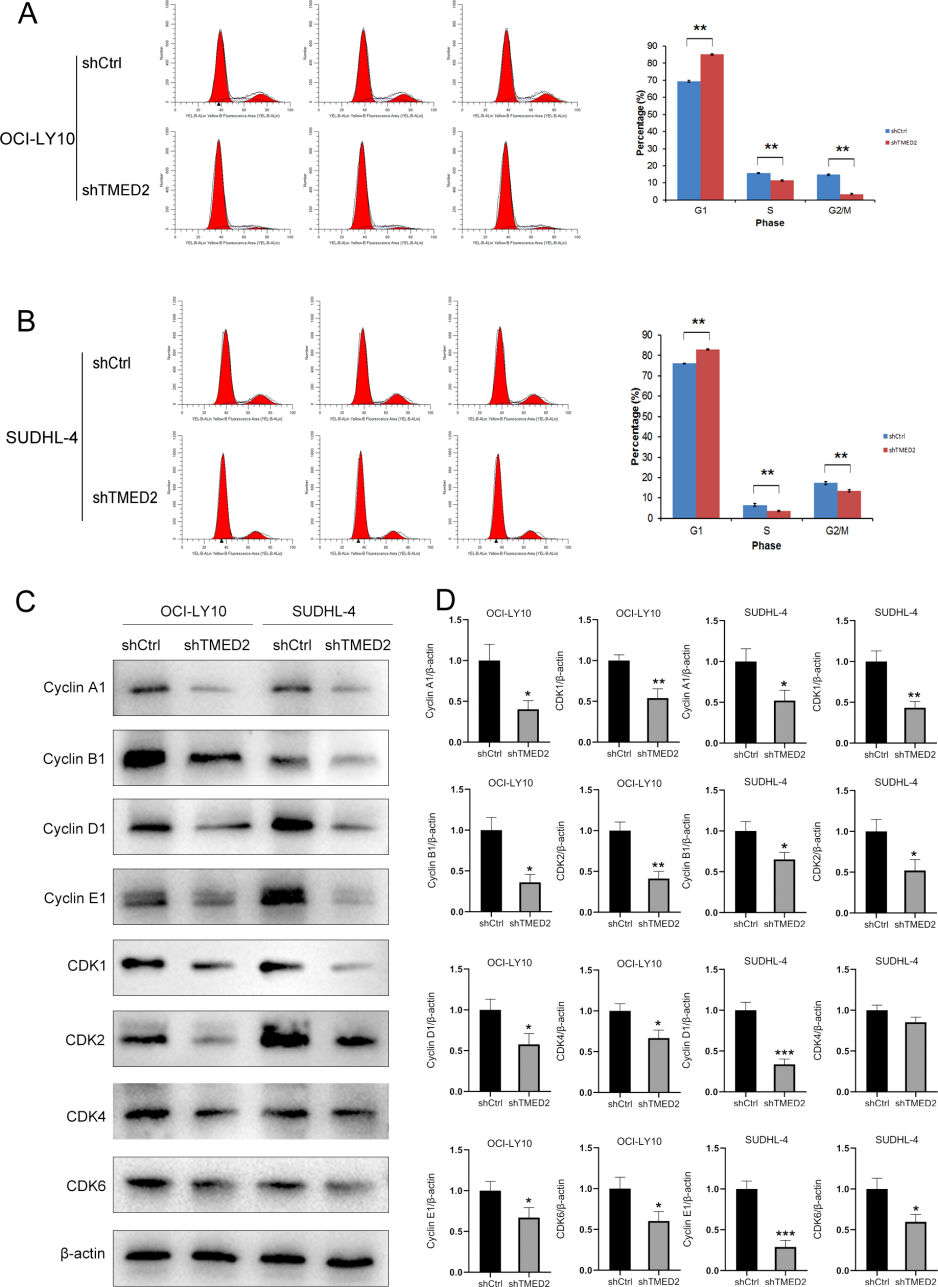
TMED2 knockdown induces G0/G1 cell cycle arrest and downregulates cell cycle regulators in DLBCL cells. **(A, B)** Flow cytometric analysis of cell cycle distribution in OCI-LY10 and SUDHL-4 cells following TMED2 knockdown (shTMED2) or control (shCtrl). Representative histograms showing DNA content (PI staining) with percentages of cells in G0/G1, S, and G2/M phases. **(C, D)** WB analysis of key G1-phase cyclins (Cyclin D1, Cyclin E1) and CDKs (CDK2, CDK4, CDK6), as well as Cyclin A1, Cyclin B1, and CDK1 in OCI-LY10 and SUDHL-4 cells after TMED2 knockdown. β-actin served as a loading control. All experiments were performed in triplicate. *p < 0.05, **p < 0.01, ***p < 0.001.

Cell cycle progression plays a crucial role in cancer cell proliferation (23). To investigate the effect of TMED2 knockdown on cell cycle distribution in OCI-LY10 and SUDHL-4 cells, we performed flow cytometry analysis. The results showed that the proportion of cells in the G0/G1 phase increased from 69.48 ± 0.61% to 85.13 ± 0.42% in OCI-LY10 cells and from 76.12 ± 0.11% to 83.02 ± 0.35% in SUDHL-4 cells. Concurrently, the percentage of cells in S phase decreased from 15.76 ± 0.31% to 11.49 ± 0.41% in OCI-LY10 cells and from 6.51 ± 0.75% to 3.59 ± 0.33% in SUDHL-4 cells. Similarly, the proportion of cells in the G2/M phase declined from 14.76 ± 0.32% to 3.38 ± 0.42% in OCI-LY10 cells and from 17.37 ± 0.70% to 13.39 ± 0.54% in SUDHL-4 cells (**Figures 3A, B**). These findings indicate that TMED2 knockdown induces G0/G1 phase arrest and suppresses cell cycle progression in both DLBCL cell lines.

To elucidate the molecular basis underlying the growth inhibitory effects of TMED2 knockdown in DLBCL cells, we examined key cell cycle regulators governing the G0/G1 phase checkpoint, including cyclin D1, cyclin E1, CDK2, CDK4, and CDK6. The cyclin D1-CDK4/6 and cyclin E1-CDK2 complexes are critical for driving the G0/G1 to S phase transition (24, 25). WB analysis revealed that TMED2 knockdown markedly reduced the protein expression levels of cyclin D1, cyclin E1, CDK2, CDK4, and CDK6 in OCI-LY10 cells, and similarly downregulated cyclin D1, cyclin E1, CDK2, and CDK6 in SUDHL-4 cells compared to the control group (**Figures 3C, D**). These findings indicate that TMED2 depletion disrupts the expression of essential G1-phase cyclins and CDKs, providing a mechanistic explanation for the observed G0/G1 cell cycle arrest. Additionally, we observed reduced expression of Cyclin A1, Cyclin B1, and CDK1 in both SUDHL-4 and OCI-LY10 cells following TMED2 knockdown (**Figures 3C, D**), suggesting a broader suppressive effect on cell cycle progression beyond the G1/S transition. Collectively, these results demonstrate that TMED2 promotes DLBCL cell proliferation, at least in part, by sustaining the expression of multiple cyclins and CDKs required for orderly cell cycle advancement.

### 3.4 Knockdown of TMED2 promoted cell apoptosis in DLBCL cells

**Figure 4:**
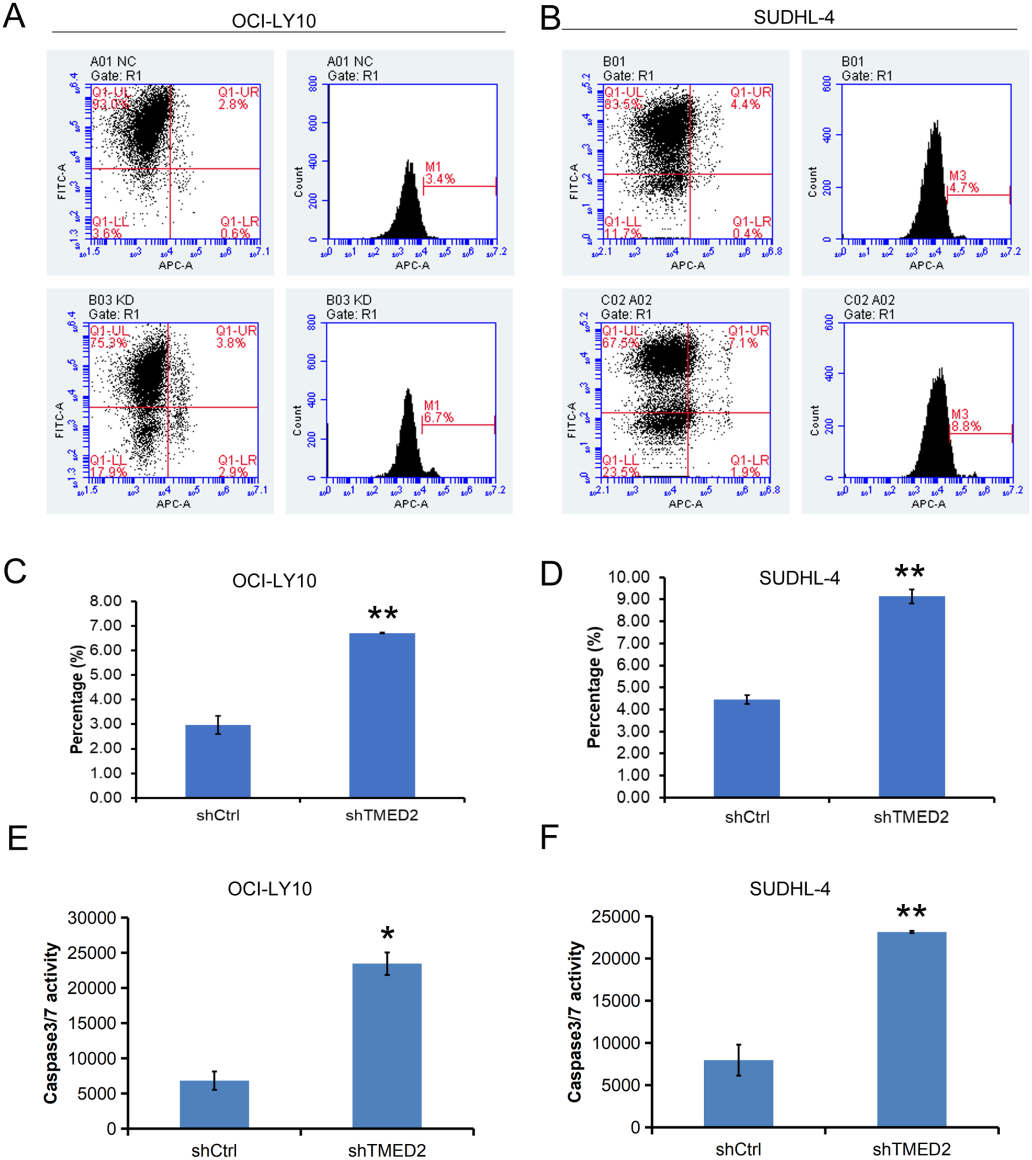
TMED2 knockdown promotes apoptosis and activates caspase-3/7 in DLBCL cells. **(A, B)** Representative flow cytometry plots of Annexin V-APC staining in OCI-LY10 **(A)** and SUDHL-4 **(B)** cells transduced with shCtrl or shTMED2. **(C, D)** Quantification of apoptosis rates in OCI-LY10 **(C)** and SUDHL-4 **(D)** cells (mean ± SD, n = 3). *p < 0.05 vs. shCtrl. Caspase-3/7 activity measured by luminescence assay in OCI-LY10 **(E)** and SUDHL-4 **(F)** cells after TMED2 knockdown. Values are expressed as fold change relative to shCtrl (mean ± SD, n = 3). All experiments were performed in triplicate. *p < 0.05, **p < 0.01 vs. shCtrl.

Next, we examined the impact of TMED2 knockdown on cell apoptosis. The Annexin V-APC flow cytometry single-staining results showed that, compared to the shCtrl group, the apoptosis rate was significantly increased in the shTMED2 group. Specifically, in OCI-LY10 cells, the apoptosis percentage was 3.4% in the shCtrl group and 6.7% in the shTMED2 group (**Figures 4A, C**). Correspondingly, in SUDHL-4 cells, the apoptosis percentage was 4.7% in the shCtrl group and 8.8% in the shTMED2 group (**Figures 4B, D**). These results indicate that knockdown of TMED2 promotes apoptosis in both tested cell lines, suggesting a potential anti-apoptotic role of TMED2 under normal conditions. The consistent increase in apoptosis across different cell models further supports the functional involvement of TMED2 in regulating cell survival.

The increased proportion of Annexin V-positive OCI-LY10 and SUDHL-4 cells following TMED2 knockdown suggests the engagement of executioner caspases in the apoptotic process. Caspase-3 and caspase-7, as key effector caspases, are activated through either the extrinsic or intrinsic apoptotic pathways and mediate the cleavage of numerous cellular substrates, leading to characteristic morphological and biochemical changes associated with apoptosis, such as chromatin condensation and DNA fragmentation (21). Notably, knockdown of TMED2 significantly enhanced caspase-3/7 activity in both DLBCL cell lines, with increases of approximately 243.8% in OCI-LY10 cells and 190.5% in SUDHL-4 cells compared to the shCtrl group (**Figures 4E, F**). These results demonstrate that TMED2 depletion promotes apoptotic cell death in a caspase-dependent manner, further supporting its role as a regulator of cell survival in DLBCL.

### 3.5 Knockdown of TMED2 downregulated protein expression of cell cycle regulators

**Figure 5:**
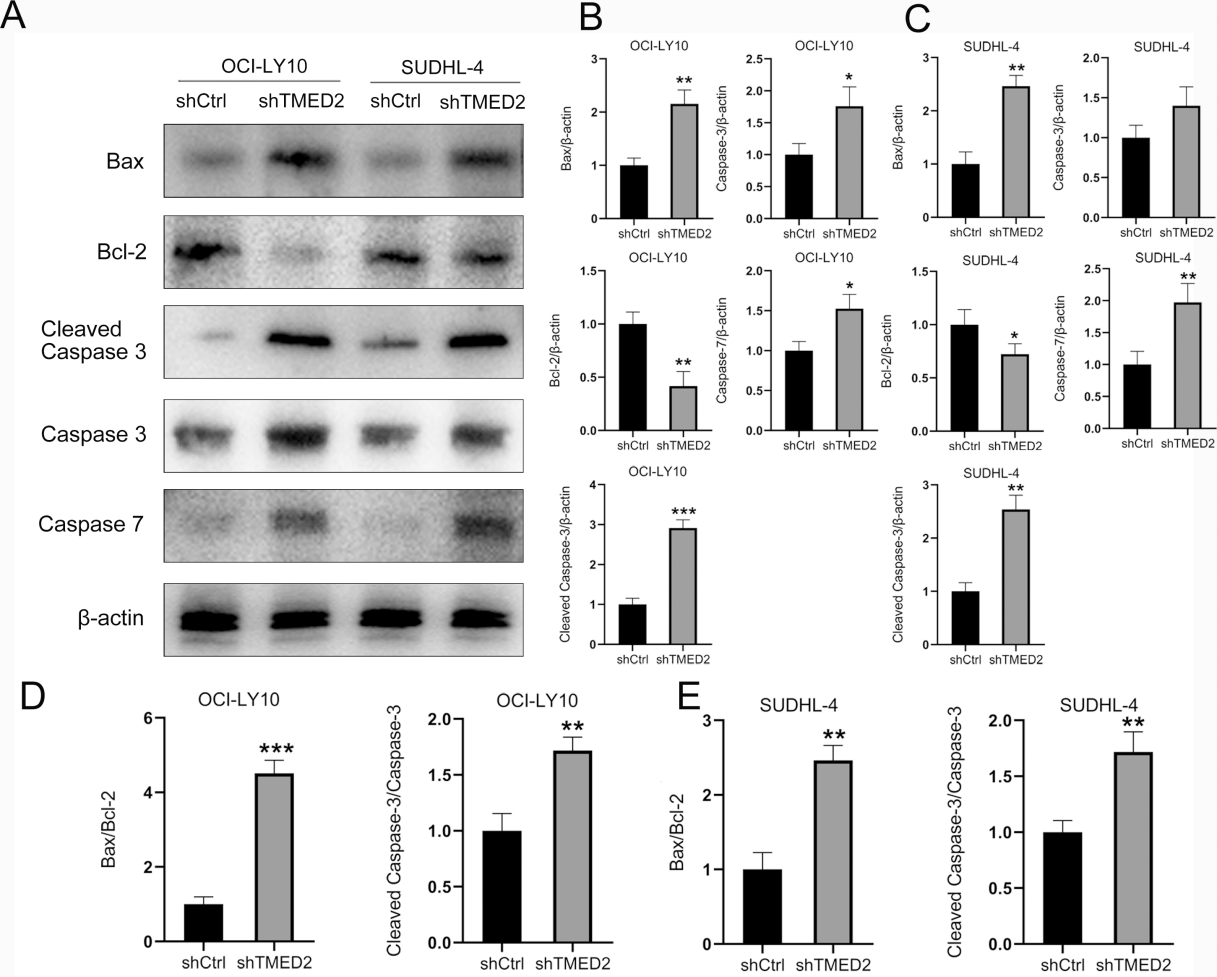
Knockdown of TMED2 promotes apoptosis in DLBCL cells by modulating caspase and Bcl-2 family protein expression. **(A)** WB analysis of caspase-3, cleaved caspase-3, caspase-7, Bcl-2, and Bax protein expression in OCI-LY10 and SUDHL-4 cells after knockdown of TMED2 (shTMED2) or control (shCtrl). β-actin served as a loading control. **(B, C)** Quantitative densitometric analysis of caspase-3, cleaved caspase-3, caspase-7, Bcl-2, and Bax protein levels in OCI-LY10 **(B)** and SUDHL-4 **(C)** cells normalized to β-actin. **(D, E)** Densitometric analysis of the ratios of Cleaved caspase 3/Caspase 3 and Bax/Bcl-2 between shCtrl or shTMED2 groups in OCI-LY10 and SUDHL-4 cells. N = 3. *p < 0.05, **p < 0.01, ***p < 0.001.

Efficient elimination of cancer cells through programmed cell death, or apoptosis, has long been a cornerstone and key therapeutic target in clinical oncology. Building on our previous findings (**Figure 4**), which demonstrated that knockdown of TMED2 significantly enhances caspase-3/7 activity in OCI-LY10 and SUDHL-4 cells, we further investigated its effect on apoptotic protein expression. Consistent with the functional activation of caspases, WB analysis revealed that TMED2 silencing markedly increased the protein levels of both caspase-3 and caspase-7 in OCI-LY10 and SUDHL-4 cells (**Figures 5A–C**). These results collectively indicate that TMED2 knockdown promotes apoptosis in DLBCL cells by augmenting both the expression and activity of key executioner caspases. Furthermore, cleaved caspase-3, the active form of caspase-3 (26), was significantly upregulated following TMED2 knockdown as demonstrated by WB analysis in OCI-LY10 and SUDHL-4 cells (**Figures 5A, D, E**).

The B-cell lymphoma 2 (Bcl-2) family is essential for regulating apoptosis, thereby influencing developmental processes, immune function, and tissue homeostasis. Dysregulation of Bcl-2 family members is frequently observed in DLBCL (27). Structurally characterized by conserved BH domains, these proteins are categorized into anti-apoptotic (e.g., Bcl-2, Bcl-xl), pro-apoptotic (e.g., Bax, Bak), and BH3-only subgroups. In this study, we evaluated how TMED2 knockdown modulates Bcl-2 and Bax expression. Bcl-2 inhibits apoptosis by suppressing caspases (including caspase-3, -6, -7, and -9) and modulating calcium signaling, thereby promoting cell survival (28). Conversely, activated Bax localizes to mitochondria, inducing membrane permeabilization and triggering apoptosis. Notably, shTMED2 increased pro-apoptotic Bax levels while reducing anti-apoptotic Bcl-2 expression (**Figures 5A–C**). The Bax/Bcl-2 ratio is recognized as a pivotal indicator for triggering apoptosis (29). In our study, WB analysis revealed that shTMED2 significantly increased the expression ratio of Bax to Bcl-2 (**Figures 5A, D, E**), suggesting that TMED2 knockdown promotes apoptosis by modulating the balance between pro-apoptotic and anti-apoptotic proteins in DLBCL cells.

### 3.6 Knockdown of TMED2 suppressed DLBCL tumor growth in tumor-bearing mice

**Figure 6:**
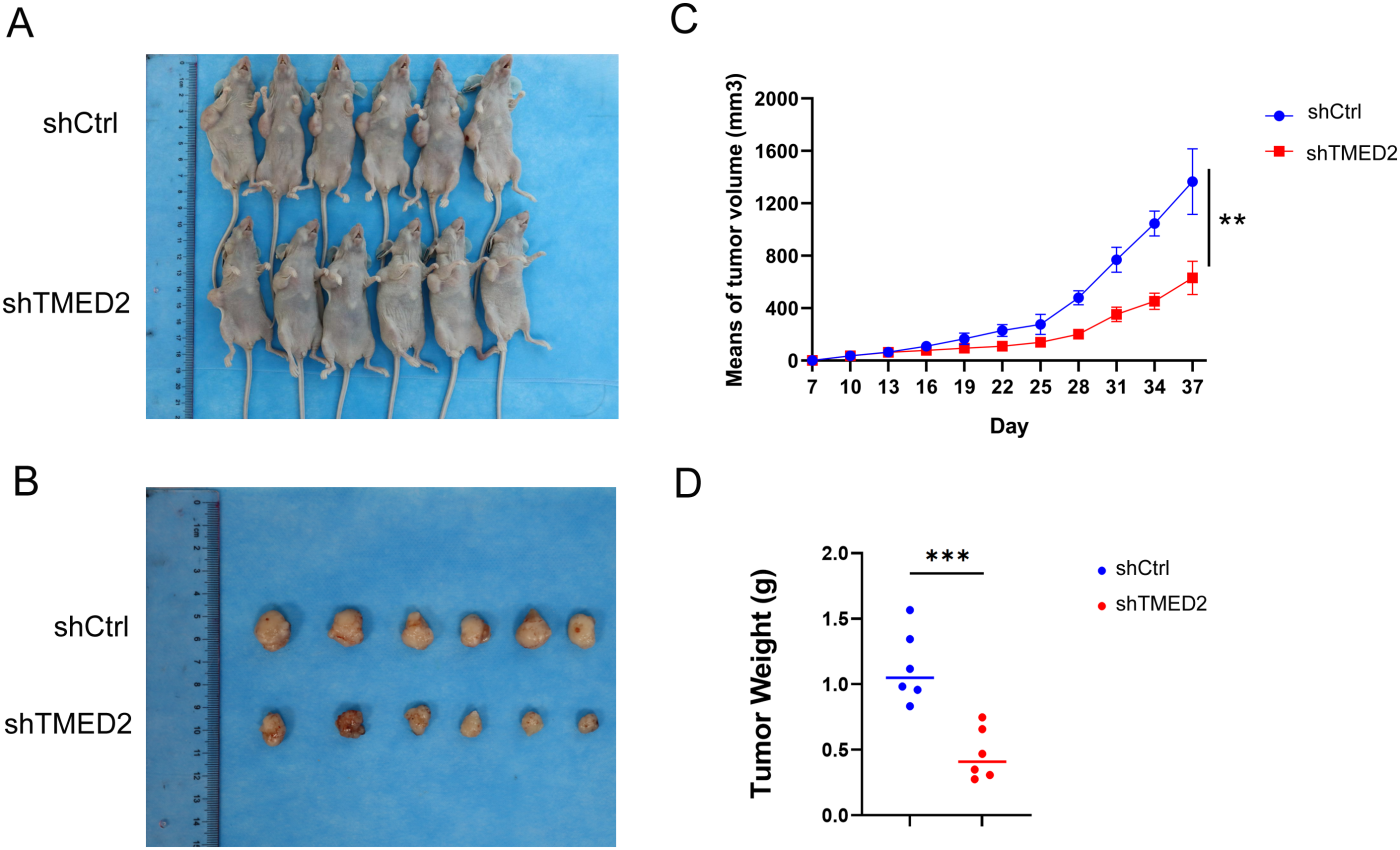
TMED2 knockdown suppresses DLBCL tumor growth *in vivo*. **(A, B)** Representative images of tumor xenografts harvested from nude mice implanted with SUDHL-4 cells stably transduced with shCtrl or shTMED2 after 37 days. **(C)** Tumor volumes were measured every 3 days in nude mice bearing subcutaneous xenografts derived from SUDHL-4 cells transduced with shCtrl or shTMED2. **(D)** Average tumor weights at the experimental endpoint, demonstrating a significant reduction in the shTMED2 group (P < 0.001). Data are presented as mean ± SD (n = 6 per group). Statistical significance was determined by Student’s t-test. **p < 0.01 vs. shCtrl. ***p < 0.001 vs. shCtrl.

To investigate the role of TMED2 *in vivo*, we established a xenograft model by subcutaneously implanting SUDHL-4 cells stably transduced with shTMED2 or shCtrl into nude mice. As shown in **Figures 6A, B**, tumor growth was markedly suppressed in the shTMED2 group compared with the shCtrl group. Significant inhibition of tumor growth rate and reduction in tumor volume became evident starting from 25 days after implantation. Over time, the difference in tumor size between the shTMED2 and shCtrl groups progressively widened, reaching its maximum at the experimental endpoint of 37 days (p < 0.001, **Figure 6C**). Consistent with the growth curves, the average tumor weight in the shTMED2 group was significantly lower than that in the shCtrl group (p < 0.001, **Figure 6D**). These results collectively indicate that silencing TMED2 effectively attenuates DLBCL tumor growth *in vivo*, reinforcing the oncogenic function of TMED2 observed *in vitro*. Together with our earlier findings that TMED2 knockdown impairs proliferation, induces G0/G1 arrest, and promotes apoptosis in DLBCL cells, the *in vivo* data further substantiate TMED2 as a critical promoter of tumor progression and a potential therapeutic target in DLBCL.

## 4 Discussion

### 4.1 Reaffirming the oncogenic role of TMED2 in DLBCL: from clinical correlation to molecular mechanism

DLBCL remains a therapeutic challenge due to its profound molecular heterogeneity. Although the R-CHOP regimen has improved outcomes, approximately 40% of patients succumb to refractory or relapsed disease, underscoring the urgent need to identify novel molecular drivers and therapeutic targets (30, 31). The primary objective of this study was to elucidate the biological function and underlying mechanisms of TMED2 in DLBCL, a protein previously implicated in vesicular trafficking but whose role in lymphomagenesis has remained obscure. Our investigation provides the first evidence that TMED2 acts as a potent oncogene in DLBCL. We demonstrated that TMED2 is significantly upregulated in DLBCL tissues and cell lines compared to normal controls, and high expression levels correlate with inferior overall survival, establishing its potential as a prognostic biomarker. Functionally, silencing TMED2 effectively suppressed tumor growth both *in vitro* and *in vivo*. Mechanistically, we identified that this suppression is mediated through a dual pathway: the induction of G0/G1 cell cycle arrest via the downregulation of critical Cyclin-CDK complexes, and the promotion of caspase-dependent intrinsic apoptosis through the modulation of the Bcl-2/Bax axis.

These findings align with emerging evidence in solid tumors, such as epithelial ovarian cancer and glioma, where TMED2 overexpression has been linked to aggressive phenotypes and poor clinical outcomes (12, 13). However, unlike previous studies that primarily focused on the secretory functions of TMED2 or its regulation of the IGF2/IGF1R/PI3K/Akt pathway in epithelial cells, our study is the first to systematically map the downstream effectors of TMED2 in a hematologic malignancy context. By pinpointing the specific disruption of the Cyclin D1/CDK4/6 and Cyclin E1/CDK2 axes, as well as the tipping of the Bax/Bcl-2 balance, our results significantly advance the understanding of DLBCL pathogenesis. This study not only validates TMED2 as a crucial promoter of DLBCL progression but also fills a critical gap in the literature regarding the non-canonical roles of p24 family proteins in B-cell malignancies.

### 4.2 Comparative analysis with existing literature: unique mechanisms of cell cycle regulation and apoptosis

Our findings regarding TMED2’s impact on cell proliferation and survival show broad consistency with its characterized role in other malignancies, yet they reveal distinct regulatory nuances specific to the lymphoma context. Previous pan-cancer analyses have suggested that TMED2 dysregulation is a common feature in glioma, breast, and ovarian cancers (12). For instance, biological studies in ovarian cancer cells demonstrated that TMED2 facilitates proliferation and migration, potentially by modulating the transport of growth factor receptors (13). Our data corroborates this proliferative advantage; however, we observed a more profound impact on the cell cycle machinery than previously reported. While studies in oral cancer have linked TMED2 to stemness via GALNT7 (14), our results in DLBCL cell lines (SUDHL-4 and OCI-LY10) highlight a direct and catastrophic failure of the G1/S transition upon TMED2 knockdown. The profound downregulation of Cyclin D1, Cyclin E1, CDK4, and CDK6 observed in our study suggests that TMED2 may be integral to stabilizing these proteins or regulating the transcription factors that drive their expression, a mechanism that appears more prominent in DLBCL than in the previously studied solid tumor models.

Furthermore, a key innovation of our study lies in the detailed dissection of the apoptotic pathways. While limited prior research hinted at TMED2’s survival-promoting capacity, our study provides robust evidence of its anti-apoptotic function through the intrinsic mitochondrial pathway. We observed a significant elevation in the Bax/Bcl-2 ratio and the subsequent activation of Caspase-3 and Caspase-7 following TMED2 silencing. Specifically, distinct from previous reports in lung adenocarcinoma where TMED2 inhibition induces apoptosis indirectly by dampening TLR4/NF-κB signaling pathway (32), or in thyroid cancer where it suppresses cell apoptosis through mTORC1-mediated fatty acid metabolism (33), our study identifies a direct engagement of the intrinsic apoptotic machinery in DLBCL through the explicit dysregulation of the Bax/Bcl-2 ratio. The direct modulation of Bcl-2 family proteins suggests that TMED2 might influence the cellular stress response or the unfolded protein response (UPR) within the endoplasmic reticulum (ER) of rapidly dividing B-cells. Given that DLBCL cells often exist in a state of chronic ER stress due to high immunoglobulin production, TMED2’s canonical role in ER-to-Golgi trafficking might be co-opted to mitigate proteotoxic stress, thereby preventing apoptosis. Thus, our study not only confirms the oncogenic nature of TMED2 seen in other cancers but also uniquely positions it as a guardian of cell cycle progression and mitochondrial integrity specifically within the high-stress environment of DLBCL.

### 4.3 Limitations of the current study: sample size, mechanistic depth, and model constraints

While our study provides compelling evidence for the oncogenic role of TMED2 in DLBCL, several limitations should be acknowledged. First, although we utilized both GCB-type (SUDHL-4) and ABC-type (OCI-LY10) cell lines to investigate the effects of TMED2 knockdown, the molecular heterogeneity of DLBCL is extensive, involving numerous genetic mutations and diverse signaling pathways beyond the cell-of-origin classification. Relying on only two cell lines may limit the generalizability of our findings to all DLBCL subtypes or rare genetic variants. Therefore, while our data convincingly demonstrate the oncogenic role of TMED2 in these specific models, broader conclusions regarding DLBCL progression across the entire disease spectrum should be interpreted with caution. Future studies incorporating a more extensive panel of DLBCL cell lines and patient-derived xenograft (PDX) models are warranted to further validate the clinical relevance and universal therapeutic potential of targeting TMED2. Second, although we established that TMED2 silencing induces G0/G1 arrest and apoptosis by downregulating Cyclin-CDK complexes and altering the Bax/Bcl-2 ratio, the upstream molecular mechanisms remain incompletely defined. The direct binding partners of TMED2 and the specific signaling cascades leading to these effects were not identified, representing a gap in mechanistic depth. Third, the *in vivo* validation relied on a subcutaneous xenograft model in immunodeficient nude mice. While this model confirmed reduced tumor growth, it does not recapitulate the systemic dissemination of lymphoma or the critical interactions within the native tumor microenvironment (TME), particularly with immune cells. Consequently, potential immunomodulatory functions of TMED2 could not be assessed. These constraints highlight the need for cautious interpretation and further investigation to fully elucidate TMED2’s role in DLBCL pathogenesis and therapy. Fourth, our *in vitro* expression analyses utilized the EBV-transformed HMy2.CIR cell line as a non-malignant B-cell control. While this immortalized line provides necessary experimental stability and serves as a recognized standard in B-cell malignancy research, it may not perfectly recapitulate the precise physiology of primary resting B cells. The immortalization process inherently alters baseline proliferation and survival signaling. However, the observation that TMED2 is still profoundly upregulated in DLBCL cells compared to this actively proliferating control underscores its specific oncogenic drive. Nevertheless, future studies should aim to validate TMED2 expression profiles and its functional mechanisms using primary human B cells isolated from healthy donors to further solidify its specific role in normal versus malignant B-cell biology. These limitations, however, do not diminish the validity of the core findings but rather highlight specific areas where the interpretation of the data should be contextualized within the scope of preclinical exploration.

### 4.4 Proposed countermeasures and methodological recommendations

To address the limitations of single-center sampling and molecular heterogeneity, future research should prioritize large-scale, multi-center cohorts with comprehensive clinical annotation, specifically incorporating established molecular classifications such as cell-of-origin (GCB/ABC) and genetic subtypes (e.g., MCD, BN2) (2, 34). This approach, potentially leveraging tissue microarrays and public genomic repositories, will enable robust validation of TMED2’s prognostic significance across diverse DLBCL subgroups and determine its independence from known risk factors.

To elucidate the incomplete upstream mechanisms, advanced molecular techniques are required. The direct protein interactome of TMED2 in DLBCL cells should be mapped using co-immunoprecipitation coupled with mass spectrometry, aiming to identify binding partners that could explain its impact on cell cycle regulators such as Cyclin D1 and CDK4/6, and apoptotic proteins. This could reveal whether TMED2 functions through direct protein stabilization, transcriptional modulation, or involvement in specific stress response pathways within the endoplasmic reticulum. Finally, to transcend the constraints of subcutaneous xenograft models, more physiologically relevant systems such as patient-derived xenograft models should be employed to assess therapeutic potential while preserving tumor-stromal interactions. To investigate potential roles in the tumor microenvironment, as suggested by broader analyses, immunocompetent or NOD/SCID mouse models would be invaluable for exploring any immunomodulatory functions of TMED2 (35). Concurrently, developing targeted pharmacological agents, such as small-molecule disruptors of TMED2 function, will be essential to translate these genetic findings into preclinical therapeutic validation and to assess potential synergies with existing chemotherapeutic agents.

### 4.5 Future directions and research outlook

Looking forward, the identification of TMED2 as a vulnerability in DLBCL opens several promising avenues for investigation. A critical area for future research is the intersection between TMED2 function and chemotherapy resistance. Since our data indicates that TMED2 silencing promotes apoptosis and cell cycle arrest, it is plausible that high TMED2 levels contribute to the resistance mechanisms against R-CHOP, particularly by raising the apoptotic threshold via Bcl-2 upregulation. Future studies should investigate whether targeting TMED2 can sensitize refractory DLBCL cells to doxorubicin or rituximab, potentially offering a rationale for combination therapies in relapsed patients.

Another exciting direction is the exploration of TMED2’s role in the unfolded protein response (UPR) and ER stress. DLBCL cells are characterized by high rates of protein synthesis; if TMED2 is essential for maintaining ER homeostasis, its inhibition could trigger “fatal” ER stress, a mechanism distinct from standard chemotherapy. Investigating markers of the UPR (such as CHOP, IRE1, or PERK) in the context of TMED2 depletion could reveal a synthetic lethal interaction with proteasome inhibitors like bortezomib. Finally, given the pan-cancer association of TMED2 with immune checkpoint expression mentioned in recent datasets (12), it is imperative to investigate whether TMED2 regulates the surface presentation of PD-L1 in lymphoma cells. If TMED2 trafficking is required for PD-L1 membrane localization, targeting TMED2 could synergize with immune checkpoint blockade therapies, thereby tackling the tumor from both intrinsic survival and extrinsic immune-evasion angles. In conclusion, this study establishes TMED2 as a novel oncogenic driver in DLBCL, and continued exploration of its multifaceted roles promises to uncover new therapeutic strategies for this difficult-to-treat malignancy.

## Data Availability

The original contributions presented in the study are included in the article/supplementary material. Further inquiries can be directed to the corresponding author.
